# Survey and Sequence Characterization of Bovine Mastitis-Associated *Escherichia coli* in Dairy Herds

**DOI:** 10.3389/fvets.2020.582297

**Published:** 2020-12-07

**Authors:** John I. Alawneh, Ben Vezina, Hena R. Ramay, Hulayyil Al-Harbi, Ameh S. James, Martin Soust, Robert J. Moore, Timothy W. J. Olchowy

**Affiliations:** ^1^Good Clinical Practice Research Group, School of Veterinary Science, The University of Queensland, Gatton, QLD, Australia; ^2^School of Veterinary Science, The University of Queensland, Gatton, QLD, Australia; ^3^Centre for Cell Factories and Biopolymers, Griffith Institute for Drug Discovery, Griffith University, Nathan, QLD, Australia; ^4^International Microbiome Centre, Cumming School of Medicine, University of Calgary, Calgary, AB, Canada; ^5^Terragen Biotech Pty Ltd., Coolum Beach, QLD, Australia; ^6^School of Science, RMIT University, Melbourne, VIC, Australia; ^7^Faculty of Veterinary Medicine, University of Calgary, Calgary, AB, Canada

**Keywords:** cattle, genomics, virulence, plasmid, comparative genomics, antibiotic resistance, whole genome sequencing

## Abstract

*Escherichia coli* is frequently associated with mastitis in cattle. “Pathogenic” and “commensal” isolates appear to be genetically similar. With a few exceptions, no notable genotypic differences have been found between commensal and mastitis-associated *E. coli*. In this study, 24 *E. coli* strains were isolated from dairy cows with clinical mastitis in three geographic regions of Australia (North Queensland, South Queensland, and Victoria), sequenced, then genomically surveyed. There was no observed relationship between sequence type (ST) and region (*p* = 0.51). The most common Multi Locus Sequence Type was ST10 (38%), then ST4429 (13%). Pangenomic analysis revealed a soft-core genome of 3,463 genes, including genes associated with antibiotic resistance, chemotaxis, motility, adhesion, biofilm formation, and pili. A total of 36 different plasmids were identified and generally found to have local distributions (*p* = 0.02). Only 2 plasmids contained antibiotic resistance genes, a p1303_5-like plasmid encoding multidrug-resistance (trimethoprim, quaternary ammonium, beta-lactam, streptomycin, sulfonamide, and kanamycin) from two North Queensland isolates on the same farm, while three Victorian isolates from the same farm contained a pCFSAN004177P_01-like plasmid encoding tetracycline-resistance. This pattern is consistent with a local spread of antibiotic resistance through plasmids of bovine mastitis cases. Notably, co-occurrence of plasmids containing virulence factors/antibiotic resistance with putative mobilization was rare, though the multidrug resistant p1303_5-like plasmid was predicted to be conjugative and is of some concern. This survey has provided greater understanding of antibiotic resistance within *E. coli*-associated bovine mastitis which will allow greater prediction and improved decision making in disease management.

## Introduction

Bovine mastitis is the major production limiting disease in the dairy industry worldwide (1–3). In addition to the negative impact on animal welfare and farm economics, the extensive use of antimicrobials to treat and manage mastitis is a major concern to public health (4). A gram-negative opportunistic environmental bacterium, *Escherichia coli* is the coliform bacteria most frequently associated with mastitis in cattle (5–7). Many high quality genomic sequences of mastitis-associated *E. coli* are publicly available (8). Genomic analyses of “pathogenic” and “commensal” strains has shown that they appear to be genetically similar. With a few exceptions, no definitive differences in virulence factors, traits, or genotypes were found specific to mastitis-associated *E. coli* (9–11). However, several identified loci (22 genes) are either enriched or implicated in mastitis-associated *E. coli*. These include the putative proteins *ymdE* and *ycdU*, 10 genes from the phenylacetic acid degradation operon (*feaR, feaB, paaFGHIJKXY*), seven genes of the ferric citrate uptake system (*fecIRABCDE*) (10) and *eprI* (11). Addition of the *fec* locus to a non-pathogenic dairy farm *E. coli* caused it to produce intramammary inflammation in dairy cows (12). Similarly, knocking out the *fec* locus in a mastitis-causing *E. coli* strain resulted in the loss of its ability to cause mastitis. Milking hygiene and wild bird transmission have been implicated as a reservoir of mastitis-associated *E. coli* (7). However, the relatively low genetic diversity between disease-associated and commensal strains indicates the issue is more complex. It is likely that mastitis-associated *E. coli* are opportunistic pathogens originating from the bovine gastrointestinal tract because the virulence-associated genes implicated in mastitis are also found in commensal *E. coli* strains (11).

Antimicrobial agents are important in the treatment of bovine clinical mastitis (13). The widespread use of antimicrobials to manage mastitis in herds (14, 15) creates selection for, and progressive spread of resistance through sharing of conjugative plasmids or pathogenicity islands (16) as is evident from the reports of antibiotic resistant *E. coli* isolates found in dairy cows with mastitis (17–19).

Whole genome sequencing of *E. coli* isolates from mastitis cases provides valuable information regarding mobile genetic elements, antimicrobial resistance (AMR), or virulence traits and can contribute to epidemiological investigations (20–22). Incorporation of this information into targeted surveillance programs that aim to improve antimicrobial stewardship can reduce the risk of AMR.

The versatility of *E. coli* genomes allows for the acquisition of different combinations of virulence factors or divergent clades that are associated with disease of various severity (10, 11, 23–27). Evidence of geographical source variation of *E. coli* isolates, particularly for *E. coli* O157, exist (28–30). However, there is paucity in the literature investigating the possible geographical disposition of the genetically diverse *E. coli* isolates associated with mastitis. Therefore, the main objectives of this study were to examine and genomically characterize and compare *E. coli* isolates cultured from dairy cows with clinical mastitis in three distinctly different geographic and climatic regions of Australia to identify antibiotic resistance, virulence factors, and mobile genetic elements which could then inform the agricultural antimicrobial stewardship programs. A secondary objective was to examine the diversity of the isolates, in particular their specific antimicrobial resistance profiles, from these different regions to identify geographic or climatic associations that may exist as a potential basis for development of more effective and sustainable dairy mastitis treatments.

## Materials and Methods

### Sample Collection

**Figure 1:**
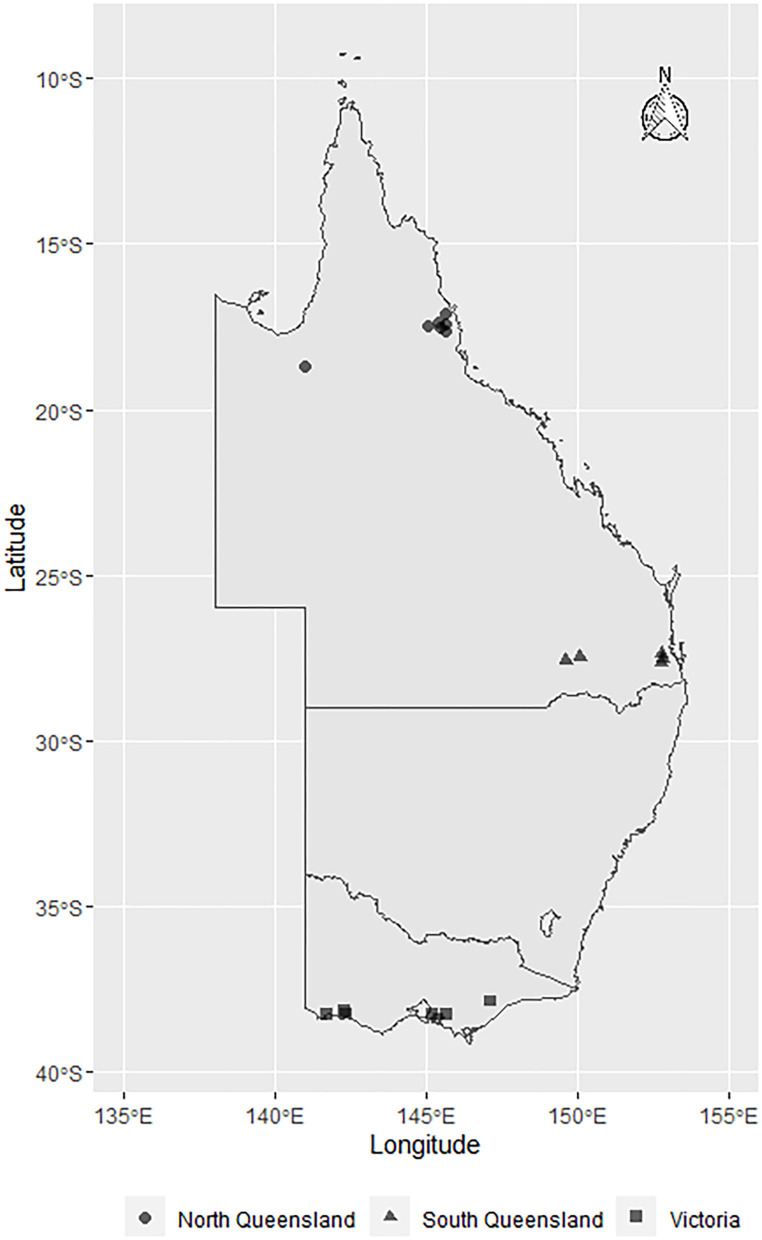
Map showing the geographical distribution of *E. coli* isolates sourced from farms across Australia: North Queensland farms (circles), South Queensland farms (triangles), and Victorian farms (squares). The symbols represent the townships centroids were the farms are located.

This cross-sectional study was conducted between March and June 2019 using milk samples (*n* = 430) collected from 29 dairy herds located in Queensland (North Queensland [NQLD], *n* = 9 herds; Southeast Queensland [SQLD], *n* = 9 herds), and Victoria ([VICT], *n* = 11 herds) (Figure 1). Herd selection was based on willingness of dairy producers to participate, ease of access to the farm location, and the cooperation of the producer's associated veterinary practice. The study was conducted in accordance with the University of Queensland Animal Ethics and National Guidelines (animal ethics approvals: SVS/ANRFA/540/18 and SVS/043/18/TERRAGEN).

Milk samples were collected from eligible dairy cows with clinical mastitis. An enrolment eligible case of clinical mastitis case was defined as an apparently healthy lactating dairy cow of any age, breed, and stage of lactation that was experiencing a new clinical mastitis at the time of enrolment in the study and had not received systemic or intramammary antimicrobials, anti-inflammatory medications, or topical treatments in the 2 weeks prior to developing mastitis. Chronic mastitis cases (apparently healthy cow with lumps palpable in the udder, and mild changes to milk) and subclinical mastitis cases (apparently healthy cow with no observable changes in the udder, and significant elevated SCC) were not eligible for enrolment. A new clinical mastitis event was defined as either the first occurrence of a mastitis event in the current lactation or a mastitis event occurring at least after 21 days following a previous mastitis event that has clinically resolved or achieved a clinical cure (31).

Milk samples were collected aseptically from individual quarters after teats were cleaned. Briefly, each sampled teat was washed dried using a single-use paper towel and the teat end scrubbed with 70% ethanol until clean. Two to three foremilk streams were discarded before ~30 mL of milk was collected into a sterile tube which was immediately capped and placed in −20°C freezer. Collected samples were delivered frozen to the veterinary microbiology laboratory of the University of Queensland for bacterial culture.

### *Escherichia coli* Isolation

Standard microbiological methods (gram stain, viable total plate count using Sheep Blood Agar (SBA, P2133 Sheep Blood Columbia Agar Plates, Thermofisher) and total viable gram negative count using MacConkey agar (MCA, PP2130 MacConkey No 3 Agar Plates, Thermofisher) were used to quantify the microorganisms present in all of the milk samples. Assessment of the haemolysis pattern (presence or absence of a clear haemolysis zone; *E. coli* 46B, 115C, and 143B were haemolytic) and biochemical tests were used to further characterize and identify the cultured bacteria. Individual colonies were sub-cultured on SBA and MCA plates and incubated aerobically at 37°C for 18–24 h. Pure isolates were then incubated in 2 mL of Brain Heart Infusion (BHI) broth, subsequently mixed with 20% glycerol and stored at −80°C. Matrix-assisted laser desorption ionization-time of flight mass spectrometry (MALDI-TOF MS; Bruker™ Daltonik, Bremen, Germany) was then used to confirm the identification.

### DNA Extraction

*E. coli* isolates were cultivated in BHI broth (37°C, orbital shaker at 300 rpm), 18–24 h. Genomic DNA was extracted using DNeasy PowerFood Microbial Kit (QIAGEN) with minor modifications. Eight mL of culture liquid was centrifuged (15 min at 4°C, 20,000 × g) to pellet the bacteria. The pellet was resuspended in 450 μL lysis buffer and incubated for 10 min at 65°C. Thereafter, the whole component was transferred to the Powerbead tubes and secured horizontally to a vortex adapter (Vortex-2 Genie®) and vortexed at a maximum speed for 10 min. After washing steps to remove protein and other inhibitors, purified DNA was eluted and the cconcentration and purity of the isolated genomic DNA evaluated using a NanoDrop ND-1000 spectrophotometer (Thermo Scientific). A sample of DNA was considered acceptable if the A260/280 ratio was ~1.8.

### Whole Genome Sequencing

The New England Biolabs NEBNext Ultra II FS DNA Library Prep Kit for Illumina for multiplexing was used according to the manufacturer's instructions (Illumina) to construct whole genome sequencing libraries. Libraries were sequenced (Illumina NextSeq 500 instrument, 300 cycle mid-output kit; 2 × 150 bp paired end) to obtain an average coverage depth of >100 and a read retention of >98%. The draft genome sequences of the 24 *E. coli* isolates have been deposited with GenBank under Bio project PRJNA644956.

### Whole Genome Assembly and Annotation

Primer sequences were removed and reads were quality trimmed using cutAdapt (32). The Nullarbor pipeline (33) was used to process the samples. SKESA assembler (version 2.3.0) (34) was used for *de-novo* assembly. Assemblies were annotated using prokka (version 1.14.5) (35) for loop with the following command.

for file in ^*^.fna; do tag=$file%.fna; prokka –prefix “$tag” –locustag “$tag” –genus Escherichia –strain “$tag” –outdir “$tag”_prokka –force –addgenes “$file”; done.

### Strain Typing

Assemblies were taxonomically assigned, Multi Locus Sequence Type (MLST) (36, 37) and scored for genome distance using Type (Strain) Genome Server (TYGS) (https://tygs.dsmz.de) (38) (date accessed: 19/5/2020). DNA-DNA hybridization values were measured based on formula 2. Pairwise comparison of genome sequences among the set of genomes were conducted by calculating precise distances using the Genome BLAST Distance Phylogeny approach (GBDP) under the algorithm “coverage” and distance formula d5 (39). These distances were used to determine the genome similarities for each of the genome pairs. 100 distance replicates were calculated each. Digital DDH values and confidence intervals were calculated using the recommended settings of the GGDC 2.1 (39).

### Pangenome Analyses

Roary version 3.13.0 (40) was used for pangenome analysis, with the following arguments.

roary -e –mafft -i 90 -v -z -s -o

This list was then annotated and characterized via screening sequences against NCBI and Gene Ontology (41) using the PANNZER2 server (42). Signal sequences were obtained from the core genome using SignalP 5.0 (Linux x86_64) (43, 44), with the following arguments.

signalp -fasta input.fasta -format short -mature -org gram- -verbose.

### Phylogenetic Analysis

A core genome alignment generated by roary was performed, including an outgroup *Escherichia fergusonii* ATCC 35469 (accession GCA_000026225.1) and ingroup *E. coli* K-12 MG1655 (accession GCF_000005845.2). RAxML (raxmlHPC-PTHREADS-SSE3 version 8.2.10) (45) was used with the following parameters, for maximum likelihood and rapid bootstrap analysis with 1,000 replicates.

raxmlHPC-PTHREADS-SSE3 -f a -x 123 -p 123 -N 1,000 -m GTRGAMMA -O -n.

The bipartitions output file was visualized in FigTree version 1.4.4 (http://tree.bio.ed.ac.uk/software/figtree/) and bootstraps visualized. Inkscape version 0.92 (https://www.inkscape.org) was used to prepare the figure.

### Plasmid Identification and Analysis

Mob-suite version 3.0.0 (46) was used to identify plasmids using the mob recon function, then visualized in ClustVis (https://biit.cs.ut.ee/clustvis/) (47). No scaling was performed on the data and clustering was performed on rows and columns using the “correlation” distance measure. Putative mobilization of plasmids was determined using the mob_typer function.

### Identification of Putative Virulence Factors

ABRicate version 1.0.1 (https://github.com/tseemann/abricate) (48) was used, along with the virulence factor database (VFDB) (49) (date accessed 1/5/20) and a custom gene list identified from the literature (Supplementary Data Sheet 3), to identify the presence of putative virulence factors in *E. coli*. Both chromosomal and plasmids were analyzed.

### Antibiotic Resistance Determination

Antimicrobial resistance prediction was performed separately on plasmid and chromosomal contigs using AMRFinderPlus version 3.8 (50). Database 2020-05-04.1 was used.

### Bacteriophage and Prophages Identification

The PHASTER (https://phaster.ca/) (51) web server was used to identify bacteriophage and prophage regions using the API tool. “Questionable” phages (score of 70–90) were grouped with “incomplete.”

### Transposon/Insertion Sequence Identification

ISESCAN version 1.7.2 (https://github.com/xiezhq/ISEScan) (52) was used to identify transposons and insertion sequences.

### Clustered Regularly Interspaced Short Palindromic Repeats (CRISPR)-Cas Identification

CRISPRCasFinder (https://crisprcas.i2bc.paris-saclay.fr/CrisprCasFinder/Index) (53) was used to identify CRISPR-Cas regions within the genomes.

### Statistical Analysis

*Fisher's* exact test was performed to identify significance in the proportion of strain types, virulence genes, and plasmids from isolates recovered from NQLD, SQLD and VICT. A two sided *p*-value obtained by Monte-Carlo simulation (*n*= 2,000) of at least 0.05 was considered to be significant. Statistical analysis was conducted using stats package implement in R (54).

## Results and Discussion

### *E. coli* Mastitis Isolates, Sequence Types, and Phylogenetic Relationships

In this multi-regional Australian study, *E. coli* bacteria were isolated from dairy cows with clinical mastitis (Figure 1). There were 10 isolates from 8 farms in VICT, 9 isolates from 7 farms in SQLD, and 5 isolated from one farm in NQLD utilized in this study.

A total of 24 isolates were sequenced and confirmed as *E. coli* (Table 1). A variety of MLST sequence types (ST) were found. The most common strain type was ST10 (38%), followed by ST4429 (13%). The frequency of ST10 within these isolates was consistent with previously published findings (11). However, not all *E. coli* ST10 are mastitis-associated *E. coli*. Some ST10 can also be isolated as commensals from the gastrointestinal tract (11). The remaining STs had only one occurrence within this dataset which reflects the degree of genetic diversity of mastitis-associated *E. coli* (11, 55, 56).

**Table 1:** *Escherichia coli* isolates used in this study.

**Isolate**	**Genome size (bp)**	**Isolate location**	**Source €**	**MLST ST ¥**	**Plasmids**
*E. coli* 1	4,938,922	Victoria	VICT1	3,476	4
*E. coli* 2	4,841,024	Victoria	VICT2	642	3
*E. coli* 3	4,724,360	Victoria	VICT3	10	4
*E. coli* 4	4,893,063	Victoria	VICT4	10	4
*E. coli* 5	4,892,754	Victoria	VICT5	10	4
*E. coli* 6	5,344,373	South Queensland	SQLD2	21	3
*E. coli* 7	4,895,802	South Queensland	SQLD3	1,123	1
*E. coli* 8	5,180,209	South Queensland	SQLD4	162	4
*E. coli* 9	4,529,954	South Queensland	SQLD5	10	0
*E. coli* 10	4,721,680	South Queensland	SQLD6	10	2
*E. coli* 19A	4,626,367	North Queensland	NQLD1	685	1
*E. coli* 20A	5,279,706	North Queensland	NQLD1	10	4
*E. coli* 20B	5,281,231	North Queensland	NQLD1	10	4
*E. coli* 21A	4,919,597	North Queensland	NQLD1	714	1
*E. coli* 21B	4,917,981	North Queensland	NQLD1	714	1
*E. coli* 46B	4,783,732	Victoria	VICT6	4,429	3
*E. coli* 55B	4,780,408	Victoria	VICT6	4,429	3
*E. coli* 55C	4,779,015	Victoria	VICT6	4,429	3
*E. coli* 69C	5,220,919	Victoria	VICT7	472	5
*E. coli* 77C	5,037,966	Victoria	VICT8	410	4
*E. coli* 111A	4,904,349	South Queensland	SQLD7	10	2
*E. coli* 111B	4,904,874	South Queensland	SQLD7	10	2
*E. coli* 115C	4,943,587	South Queensland	SQLD7	6,445	4
*E. coli* 143B	4,671,826	South Queensland	SQLD8	154	1

*ST Strain Type; € Coded farm reference identifier; ¥ MLST generated by TYGS*.

**Figure 2:**
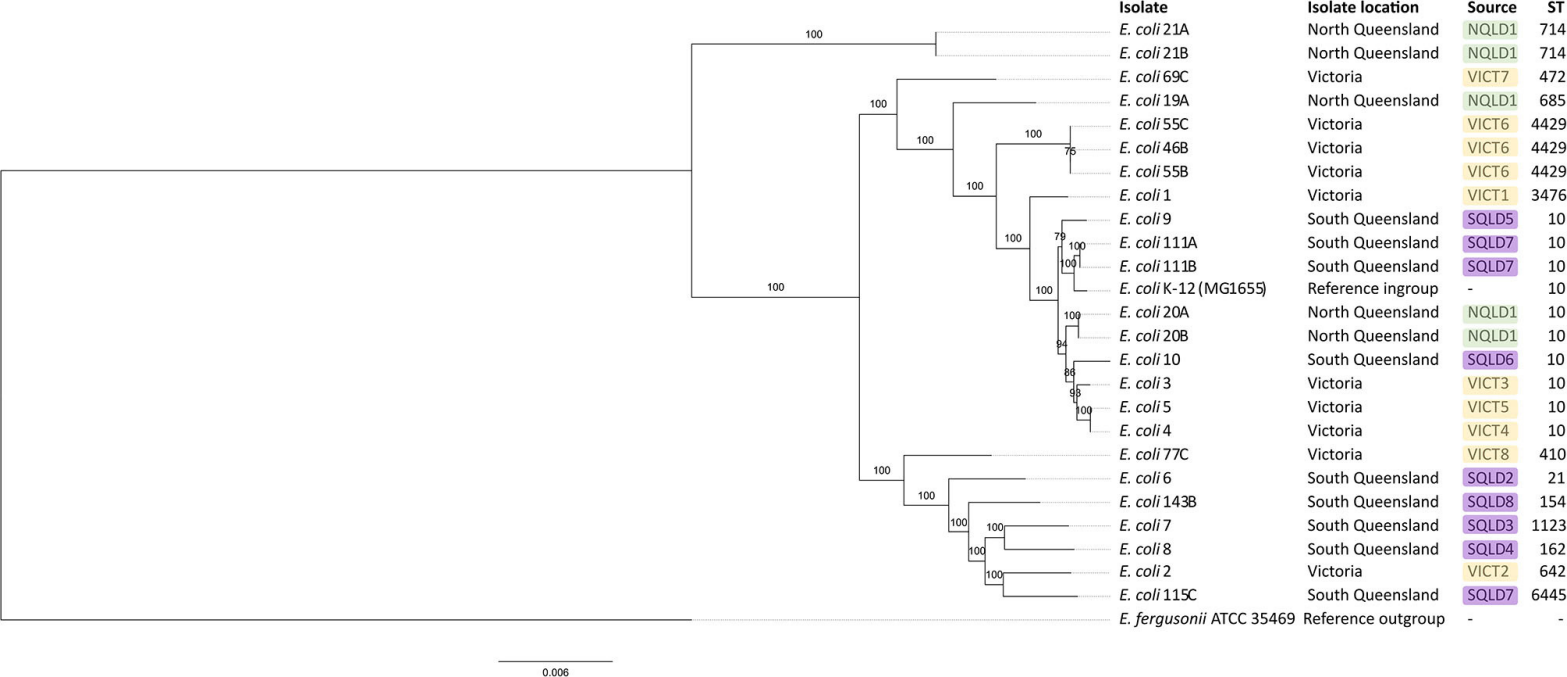
Maximum likelihood phylogenetic tree based on core genome of *E. coli* isolates identified in this study. *Escherichia fergusonii* ATCC 35469 was used as an outgroup and *E. coli* K-12 (MG1655) was used as an ingroup. Bootstrap values are shown on branches. Green labels show North Queensland isolates. Purple labels show South Queensland isolates. Yellow labels show Victorian isolates. ST stands for sequence type. Core genome alignment was performed in roary and maximum likelihood tree was built in RAxML with 1,000 replicates. Tree visualized in FigTree.

A phylogenetic tree based on a core genome alignment was generated (Figure 2). The high bootstrap values reflect the robustness of the *E. coli* MLST scheme and the well-clustered strain types. There was no relationship between ST and location (*p* = 0.51) with ST10 being found in all study regions. *E. coli* isolates from within the same farm tended to be clonal as they produced identical alignments. This is evident at VICT6, NQLD1, and SQLD7. It is notable that VICT4 and VICT5 were also identical, despite being isolated from different farms. There was no notable clustering between NQLD, SQLD, and VICT (Figure 2). This finding is further supported by the genome-to-genome comparisons (Table 2). Genome-to-genome comparisons were made for all isolates according to their genetic properties or pairs from one region. The calculation of the intergenomic distance and DNA-DNA hybridization (Table 2) showed that most genomes belong to the same genomic subspecies (results not shown). Lower DNA-DNA hybridization values can be accounted for due to presence of a diverse accessory genome, (Table 3). Isolates NQLD 19A, NQLD 20A, VICT 1, VICT 2, VICT 4, VICT 10, VICT 46B, VICT 55C, VICT 69C, VICT 77C, SQLD 5, SQLD 6, SQLD 7, SQLD 8, SQLD 21B, and SQLD 1438 were similar but slightly spaced from each other.

**Table 2:** Genome-to-genome comparison; pairwise analysis of *Escherichia coli* genomes.

**Query isolate**	**Comparator isolate**	**dDDH (d4, in %)**	**C.I. (d4, in %)**	**G+C content difference (in %)**
*E. coli* 19A	*E. coli* 20A	84.4	81.2–87.1	0.55
*E. coli* 19A	*E. coli* 46B	80.0	76.5–83.0	0.47
*E. coli* 19A	*E. coli* 8	84.1	80.8–86.8	0.43
*E. coli* 19A	*E. coli* 111A,B	87.7	84.7–90.2	0.30
*E. coli* 19A	*E. coli* 77C	86.6	83.5–89.2	0.31
*E. coli* 19A	*E. coli* 1	88.1	85.1–90.5	0.31
*E. coli* 19A	*E. coli* 2	86.4	83.3–89.0	0.34
*E. coli* 19A	*E. coli* 4	89.5	86.7–91.8	0.34
*E. coli* 19A	*E. coli* 5	89.5	86.7–91.8	0.34
*E. coli* 20A	*E. coli* 10	87.9	84.9–90.4	0.56
*E. coli* 20A	*E. coli* 21A	78.3	74.9–81.4	0.44
*E. coli* 20A	*E. coli* 115C	86.5	83.4–89.1	0.41
*E. coli* 20A	*E. coli* 7	85.5	82.3–88.2	0.40
*E. coli* 20A	*E. coli* 46B	87.0	84.0–89.6	0.39
*E. coli* 20A	*E. coli* 55C	87.0	84.0–89.6	0.39
*E. coli* 20A	*E. coli* 9	89.3	86.5–91.6	0.38
*E. coli* 20A	*E. coli* 4	86.2	83.1–88.8	0.32
*E. coli* 20A	*E. coli* 69C	74.9	71.4–78.1	0.34
*E. coli* 46B	*E. coli* 10	81.7	78.3–84.6	0.30
*E. coli* 55C	*E. coli* 10	81.7	78.3–84.6	0.30
*E. coli* 77C	*E. coli* 21B	86	82.9–88.6	0.32
*E. coli* 8	*E. coli* 21B	90.1	87.3–92.3	0.31
*E. coli* 8	*E. coli* 143B	90.2	87.4–92.4	0.30

*DDH, DNA-DNA hybridization; CI, confidence interval, d, distance*.

**Table 3:** Pangenome Analysis showing the total number of genes and percent of total pangenome.

**Pangenome breakdown**	**Designation**	**Number of genes**	**Percentage of pangenome%**
Core genes	(99% <= strains <= 100%)	3,305	33.60
Soft core genes	(95% <= strains <99%)	158	1.61
Shell genes	(15% <= strains <95%)	1,653	16.80
Cloud genes	(0% <= strains <15%)	4,721	47.99
Total genes	(0% <= strains <= 100%)	9,837	100.00

### Pangenome Analysis, Gene Content, and Chemotaxis

To uncover putative virulence factors in genes shared by all isolates, pangenomic analysis was performed. A pangenome of 9,837 genes was found, with a core genome of 3,305 genes (27.69%) (Table 3). This was consistent with previous studies of mastitis-associated *E. coli* which found 3,492 core genes from 66 isolates (10), 3,842 core orthologous groups from eight isolates (11), 1,976 genes from 20 isolates (55), and six fecal isolates (with a 70% rather than 90% cut-off) (11).

**Figure 3:**
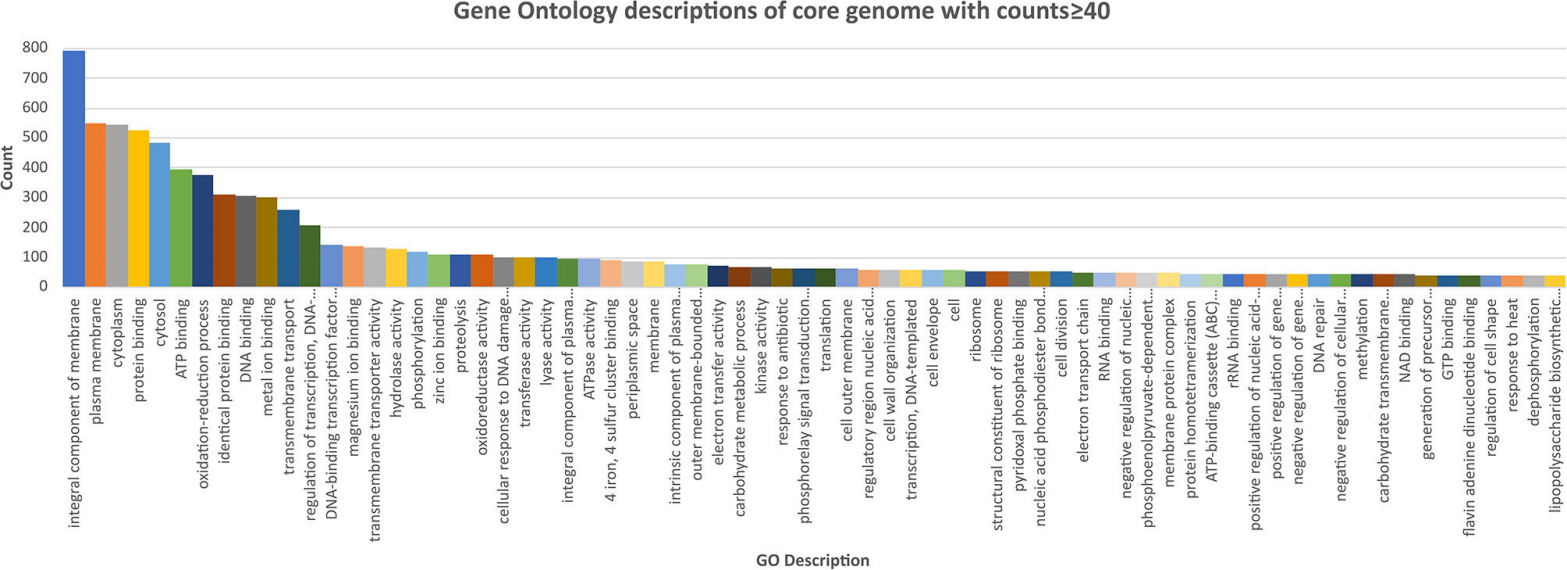
Chart of the most abundant Gene Onology (GO; core genome with counts ≥40) descriptions of the core genome of the *E. coli* isolates, ranked highest to lowest. Further GO annotations can be found in Supplementary Data Sheet 6.

The core and soft core genome (3,463 genes) were further analyzed to identify any notable characteristics. Of the soft core genome, 3,166 out of 3,463 genes (91.4%) could be assigned a Gene Ontology (GO) identity and descriptors. This reflected the high degree to which *E. coli* has been studied and characterized. The most abundant localization of proteins appeared to be an integral component of the membrane (790), followed by cytoplasm (544) (Figure 3, Supplementary Data Sheet 6).

Several genes were worthy of note. These were genes associated with response to antibiotics (74), chemotaxis (13), bacterial-type flagellum-dependent cell motility (28), cellular adhesion (17), biofilm formation (11) and pilus (14). No microcins, colicins or other bacteriocins were identified in the pangenome. Out of the soft core genome, 452 were predicted to be secreted (Supplementary Data Sheet 6), 37 were associated with proteolysis, 15 with cell adhesion, 14 with pilus, and 8 with heme binding. To further investigate the behavior of mastitis-associated *E. coli*, the core genome was screened for chemotaxis-associated genes. The screening included genes encoding maltose/maltodextrin binding (*malE*), flagellar genes (*fliGJLMNO*), signal transducer for aerotaxis sensory (*aer*), D-galactose/D-glucose-binding (*mglB*), heme binding dipeptide ABC transporter (*dppA*), NAD-dependent protein deacylase/protein desuccinylation (*cobB*), D-ribose substrate-binding (*rbsB*), methyl-accepting chemotaxis protein II (*tsr*), methyl-accepting chemotaxis protein (*trg*), and acetyl-coenzyme A synthetase (*acs*). A negative chemotaxis nickel-binding gene (*nikA*) was also identified. These chemotaxis-associated-genes may play a role in the pathogenesis of mastitis-associated *E. coli* and partially explain the presence of *E. coli* within the mammary glands. D-galactose and glucose are the components of lactose, the main sugar present in bovine milk, suggesting a beneficial role for the *mglB* gene. Similarly, the presence of the *aer* aerotaxis gene is consistent with *E. coli*'s preference of oxygen (presumably at higher levels when compared to the relatively anaerobic bovine gastrointestinal tract) which may also make milk and the mammary gland a favorable bacterial environment. The presence of *dppA*, (encodes a heme-binding protein) in combination with the core hemolysin gene (*ytjB*) may favor a tissue environment (over the gastrointestinal tract) because of the proximity to hemoglobin-containing red blood cells.

### Plasmids, Mobility, Virulence, and Antimicrobial Resistance

**Figure 4:**
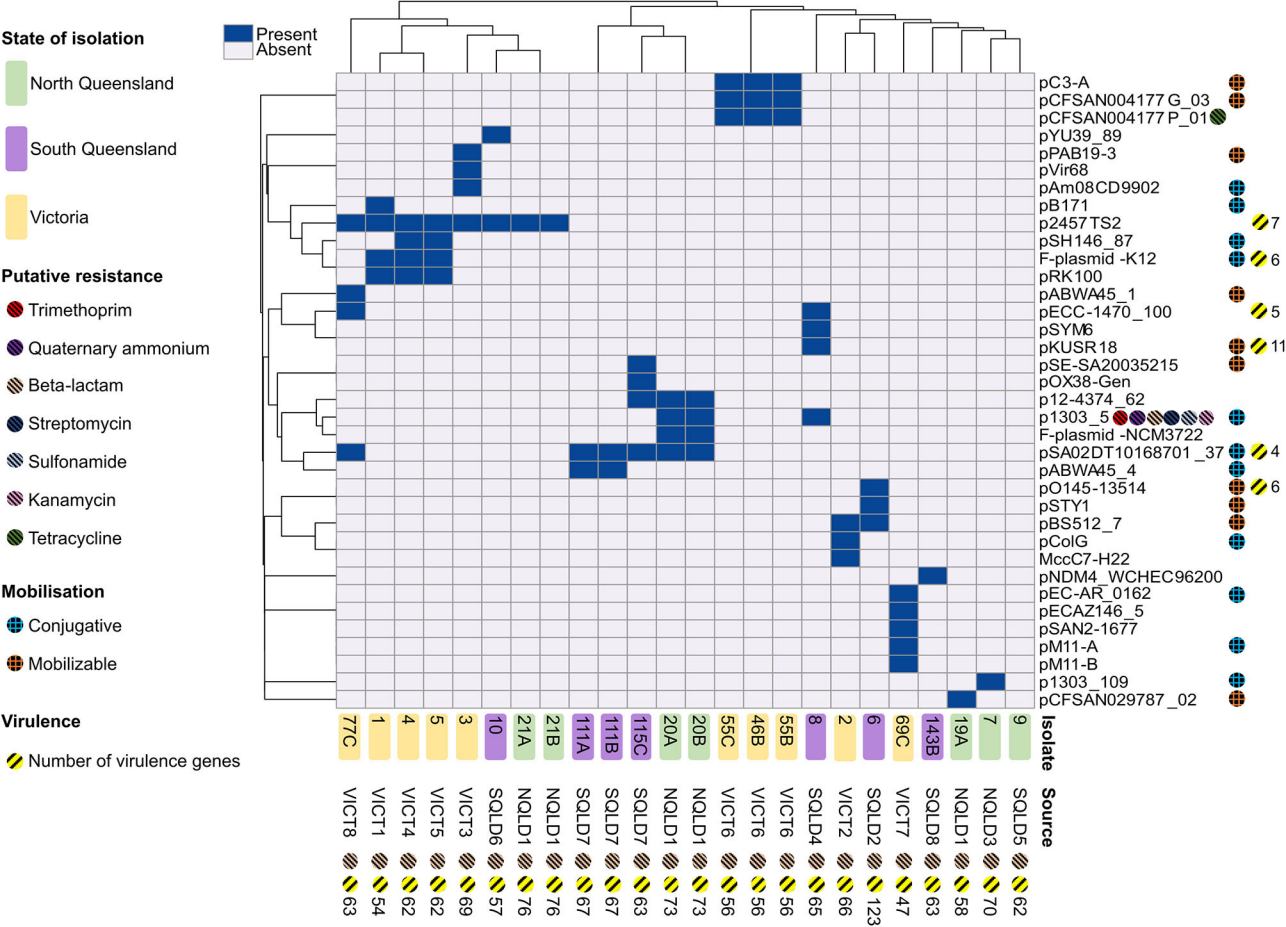
Heatmap of plasmid presence/absence within each *E. coli* isolate based on the correlation between isolates (x-axis) and plasmids (y-axis). Dark blue rectangles indicate presence of a corresponding -like plasmid. Isolate 9 contained no plasmids and was excluded from the heatmap. Victorian isolates are colored in yellow, North Queensland in green, South Queensland in purple. Plasmid elements are shown on the right y-axis. Chromosomal elements are shown along the x-axis. Putative resistance genes are shown for plasmids and chromosomal DNA for each isolate, represented by striped circles; red shows trimethoprim, purple show quaternary ammonium, peach shows beta-lactam, dark blue shows streptomycin, light blue shows sunfonamide, pink shows kanamycin, green shows tetracycline. Putative plasmid mobilization represented by hashed circles; blue shows putatively conjugative, orange shows putatively mobilizable. Yellow-striped circles and the corresponding number represent presence and number of putative virulence factors. Heatmap produced using ClustVis.

A total of 36 different plasmids were identified based on mash designations from mob-suite (Figure 4). The plasmid distribution pattern was associated with geographical location (*p* = 0.02). Of the 36 plasmids, 50% (18/36) were unique to VICT isolates, 28% (10/36) unique to SQLD, and 6% (2/36) unique to NQLD. A number of plasmids were shared between study regions: 6% between VICT and SQLD (2/36), 6% between VICT, SQLD and NQLD (2/36), and 6% between SQLD and NQLD (2/36). Supplementary Data Sheet 1 contains further information along with accession numbers of the most similar plasmids. Bacterial strains contained 0–5 plasmids, with *E. coli* 9 containing no plasmids, and *E. coli* 69C containing 5. The origins of the plasmids varied: 26 were *E. coli* plasmids, 8 were *Salmonella enterica* plasmids, 1 was a *Shigella flexneri*, and 1 was a *Shigella boydii* plasmid.

The most commonly identified plasmid (p2457TS2) was found in eight isolates across Victoria and Queensland. In general, there appeared to be a local geographical relationship between the specific isolation farm of a strain and the plasmids it carried (*p* < 0.001). Isolates from VICT6 all shared the same three plasmids. Two of four isolates from the NQLD1 farm shared four plasmids. Victorian isolates from VICT6, VICT 7, and VICT 8 shared three plasmids, and four out of VICT1–VICT5 isolates contained the p2457TS2 plasmid. VICT4 and VICT5 both shared the same four plasmids, despite being from different farms.

Plasmids were examined for putative virulence factors by screening against the VFDB and a custom database (Supplementary Data Sheet 3) created from previous genomic analyses (10–12). Putative virulence factors were present in 16.67% of plasmids (Table 4). A notable feature found in *E. coli* isolates *E. coli* 4, *E. coli* 5, *E. coli* 6, and *E. coli* 77C was the presence of RTX toxin cluster which included the hemolysin genes *hlyABCD*. HlyA has been shown to enhance the pathogenicity of extra-intestinal *E. coli* (ExPEC). A similar RTX toxin, TosA, has been implicated in uropathogenic *E. coli* (UPEC) (9, 55, 57). It is therefore possible that the RTX cluster present in these isolates is associated with or directly produces a cytotoxic effect in the mammary gland leading to tissue injury, damage and subsequently to the clinically observed signs of peracute mastitis observed in dairy cows infected by this strain. *E. coli* 4 contained two plasmids (p2457TS2-like and F-plasmid-K12-like) which contained virulence genes *fecIRABDE*; *yjhV*, and *faeCDEFHI*.

**Table 4:** Summary selection of virulence factors from bovine mastitis-associated *E. coli* isolated in this study.

**Virulence factor gene/operon**	**Function/description**	**Number of isolates (%)**	Location^*^
*CsgBDFG*	Curli biogenesis	24 (100%)	Chromosomal
*entABCDEFS*	Enterobactin biosynthesis	24 (100%)	Chromosomal
*espR1*-*espX4*-*espX5*	Non-LEE-encoded type III secreted effector	24 (100%)	Chromosomal
*fepABCDG*	Ferri-enterobactin transport	24 (100%)	Chromosomal
*Fes*	Ferri-enterobactin utilization	24 (100%)	Chromosomal
*fimFGH*	Fimbriae biosynthesis, adhesion	24 (100%)	Chromosomal
*ompA*	Outer membrane protein A	24 (100%)	Chromosomal
*paaFGHIJKXY*	Aerobic catabolism of phenylacetic acid	23 (96%)	Chromosomal
*espL1*	Predicted non-LEE-encoded type III secreted effector	22 (92%)	Chromosomal
*fimABCEI*	Type I fimbrae/pilus biosynthesis	22 (92%)	Chromosomal
*feaBR*	Phenylacetic acid degradation operon	22 (92%)	Chromosomal
*eprI*	Predicted type III secretion protein	22 (92%)	Chromosomal
*fimD*	Outer membrane usher protein, type 1 fimbrial synthesis	21 (88%)	Chromosomal
*espX1*	Predicted non-LEE-encoded type III secreted effector	20 (83%)	Chromosomal
*fdeC*	Intimin-like adhesin	20 (83%)	Chromosomal
*yjhV*	KpLE2 phage-like element	8 (33.3%)	p2457TS2
*fecIRABDE*	Ferric dicitrate transport	8 (33.3%)	p2457TS2
*hlyABCD*	Hemolysin	7 (29.2%)	pSA02DT10168701_37; pO145-13514
*faeCDEFHI*	Fimbrial protein A biogenesis	3 (12.5%)	F-plasmid-K12
*hylC*	Acylation of HylA	2 (8.3%)	pECC-1470_100
*f17d-ADG*	Periplasmic protein	2 (8.3%)	pECC-1470_100
*cnf1*	Cytotoxic necrotizing factor 1	2 (8.3%)	pECC-1470_100
*gspCDEFGHIJKLM*	Type II secretion structural biogenesis	1 (4.2%)	pKUSR18
*espP*	Extracellular serine protease	1 (4.2%)	pO145-13514
*toxB*	Toxin	1 (4.2%)	pO145-13514

*All plasmid-associated virulence factors and most abundant chromosomal virulence factors were included for brevity. Supplementary Data Sheet 1 contains further plasmid details including accession numbers, while the full hit/miss table of every isolate and virulence factor panel can be found in Supplementary Data Sheet 2*.

* *Chromosomal or plasmid. For plasmids, closest -like plasmid was described*.

Only a few antibiotic resistance genes were found in these isolates, with the exception that all isolates contained the beta-lactam resistance gene *blaEC* within their chromosome (Figure 4, Supplementary Data Sheet 2). Antibiotic resistance genes were located primarily in plasmids which correlated with the accessory genome from the pangenomic results. Two plasmids were identified with antimicrobial resistance genes, the p1303_5-like and the pCFSAN004177P_01-like plasmids. The p1303_5-like plasmid was found in two isolates from NQLD1 in North Queensland. It is a multi-drug resistant plasmid, containing trimethoprim, quaternary ammonium, beta-lactam, streptomycin, sulfonamide and kanamycin resistance genes. The pCFSAN004177P_01-like plasmid was found in 3 isolates from VICT6 in Victoria and contained a tetracycline resistance gene. With consideration to this limited dataset, the pattern is consistent with a local spread of antibiotic resistance through plasmids of bovine mastitis cases.

This analysis was limited by the small sample size. Larger sampling may uncover geographical relationships, if any exist. Furthermore, the proposed larger study should be combined with long-read genomic sequencing to allow analysis of complete plasmids. Plasmid lineages could be directly analyzed with less ambiguity.

Conjugative plasmids contain all the genes required for self-transfer. Whereas, mobilizable plasmids only contain a portion of these components, such as relaxosomal components oriT comprised of a relaxase gene and nicking proteins (58). In the presence of conjugative plasmids, mobilizable plasmids can piggyback off conjugative machinery to spread throughout a population.

To examine this possible mechanism of spread, plasmids were screened for presence of putative conjugative and mobilizable elements to predict potential mobility. Eleven (11) plasmids were conjugative, 10 were mobilizable, and the remaining 15 were non-mobilizable (Figure 4). Mobilizable plasmids were geographically clustered (*p* < 0.03). Four plasmids from six isolates were unique to VICT, three plasmids from three isolates were unique to SQLD while two plasmids from two isolates were unique to NQLD. Of all the *E. coli* isolates, 7 (29.17%) contain both putative conjugative and mobilizable plasmids which could allow the spread of mobilizable plasmids throughout the microbial community (Supplementary Data Sheet 1). If conjugative plasmids are able to spread throughout the communities and are acquired by strains which also contain mobilizable plasmids, this may assist in the communal dissemination of these mobilizable elements. Ongoing strain isolation and genomic surveillance should be undertaken to monitor this relationship and the potential risk.

Plasmid mobility did not co-occur with putative antibiotic resistance genes or virulence plasmids, except in 5/36 plasmids. However, these plasmids were found in a total of 10/24 isolates, indicating 41.7% contained mobilizable plasmids with associated virulence factors. The pKUSR18-like plasmid was putatively mobilizable and contained 11 virulence factors, found in a single isolate (*E. coli* 8) (Figure 4).

The p1303_5-like plasmid is of particular note. It is a multidrug resistant plasmid carrying six different antibiotic resistance genes and conjugative elements which creates the potential for spread throughout a population. This plasmid was found in 2 of 5 NQLD1 isolates and in the SQLD4 isolate suggesting that spread was occurring. Those 3 isolates contained multiple antibiotic resistance and more virulence genes compared to isolates which carried a single antibiotic resistance gene, a finding compatible with the previous studies (59–61). Moreover, tetracyclines and sulphonamides are preferred antibiotics for treatment of mastitis caused by gram-negative pathogens (62, 63). Internationally, it has been shown in Turkish cow herds where mastitis is managed with the fluoroquinolones (danofloxacin and enrofloxacin), *E. coli* was shown to have notable resistance to these antibiotics (61). In China, mastitis-associated *E. coli* have been shown to have notable resistance to sulfamonomethoxine and sulfamethoxazole, both used in management of intestinal infections (19). In contrast, within USA, antibiotic resistance has been shown to not correlate with antibiotic usage between 1985 and 1987 compared to 2009 (15).

Therefore, this observation may be associated with the widespread use of these antibiotics as part of a generic approach to the treatment of mastitis in cows, or farms not practicing herd culling (62–64), which may explain the presence of this multidrug resistant plasmid. However, 3 out of 5 NQLD isolates did not contain the plasmid. There may be an evolutionary disadvantage and energy burden associated with maintaining and expressing genes from this large 270,000 bp plasmid. In addition, it is also possible not every member of the microbial community must carry this plasmid for its effect to be mutually beneficial to the p1303_5-negative strains (65, 66). The pSA02DT10168701_37-like plasmid was both putatively conjugative and contained 4 virulence factors. It was found in 25% of all isolates, 3/3 from the SQLD7 farm, 2/4 from the NQLD1 farm and 1/1 from VICT8. The pO145-13514-like plasmid was putatively mobilizable and contained six virulence factors.

Antibiotic resistance-containing non-mobilizable plasmids such as the tetracycline resistance pCFSAN004177P_01-like plasmid was found in three isolates from the VICT6 farm, confirming clonality between these 3 isolates. Management of mastitis at VICT6 included a number of first-line antimicrobial treatments (62–64) providing selection pressure likely to be the responsible for the spread of the tetracycline resistance pCFSAN004177P_01-like plasmid at this farm (17–19). *E. coli* are frequently associated with mobile genetic elements containing AMR genes that have the potential to carry resistance to antimicrobials that are of importance in human medicine (67). As a member of the *Enterobacteriaceae* family, *E. coli* are omnipresent bacteria capable of rapidly mounting resistance to almost all first-line antibiotics (68). Our results are consistent with published studies on *Enterobacteriaceae* isolated from cattle (17, 19) and likely stem from widespread use or misuse of antimicrobials (26, 27, 69), selection pressure or horizontal gene transfer from intrinsically resistance commensals (19, 70).

The most common and also putatively non-mobilizable p2457TS2-like plasmid (8/24 isolates) contained the *fec* locus which consisting of 7 putative virulence factors. Knockout studies in a bovine mastitis disease model have demonstrated the *fec* locus to be an essential virulence factor (12). It is noteworthy that when the *fec* locus was not identified in a plasmid, it was detected in the chromosome of 10 isolates, a population representing 75% of all isolates. It is possible this locus is not required by every isolate, and mosaic presence within a community may be enough to cause disease.

### Chromosomal Virulence Factors

Previous comparative genomic analysis concluded highly similar virulence factor profiles are present in both mastitis-associated *E. coli* isolates and the commensal *E. coli* present in the enteric microflora. The finding indicates these genes provide a selective advantage in the gastrointestinal tract and defines mastitis-associated *E. coli* as an opportunistic pathogen (11). To examine the isolates identified in this study, chromosomes were screened for putative virulence factors using the VFDB and a custom database (Supplementary Data Sheet 3).

Twenty-four genes across 8 chromosomal loci were identified as core virulence factors (Table 4). This included the *csgBDFG, entABCDEFS, espR1-espX4-espX5, fepABCDG, fes, fimFGH*, and *ompA* genes. *fimFGH* genes are associated with epithelial cell adherence. However, knocking out *fim*H demonstrated it is important but not essential in the adherence of *E. coli* (71). The chromosomal *paaFGHIJKXY* locus was present in 23/24 strains. It was absent from *E. coli* 9, which was also the only strain to not contain a plasmid. Virulence factors *fimABCDEI* and *espL1- espX1* were found in ≥92% of strains. Previously implicated mastitis-associated virulence factors, *feaBR* (10) and *eprI* (11) were found in 92% of isolates.

Isolate *E. coli* 6 contains the most virulence factors, at 129, including 6 found on the putatively mobilizable pO145-13514-like plasmid, while *E. coli* 69C contains the least at with 47 (Supplementary Data Sheets 1, 2 contain the full virulence factor hit-miss tables). On average, isolates contained 70.25 ± [standard deviation; σ = 15.42] putative virulence factors.

### Prophages and Mobilizable Elements

**Figure 5:**
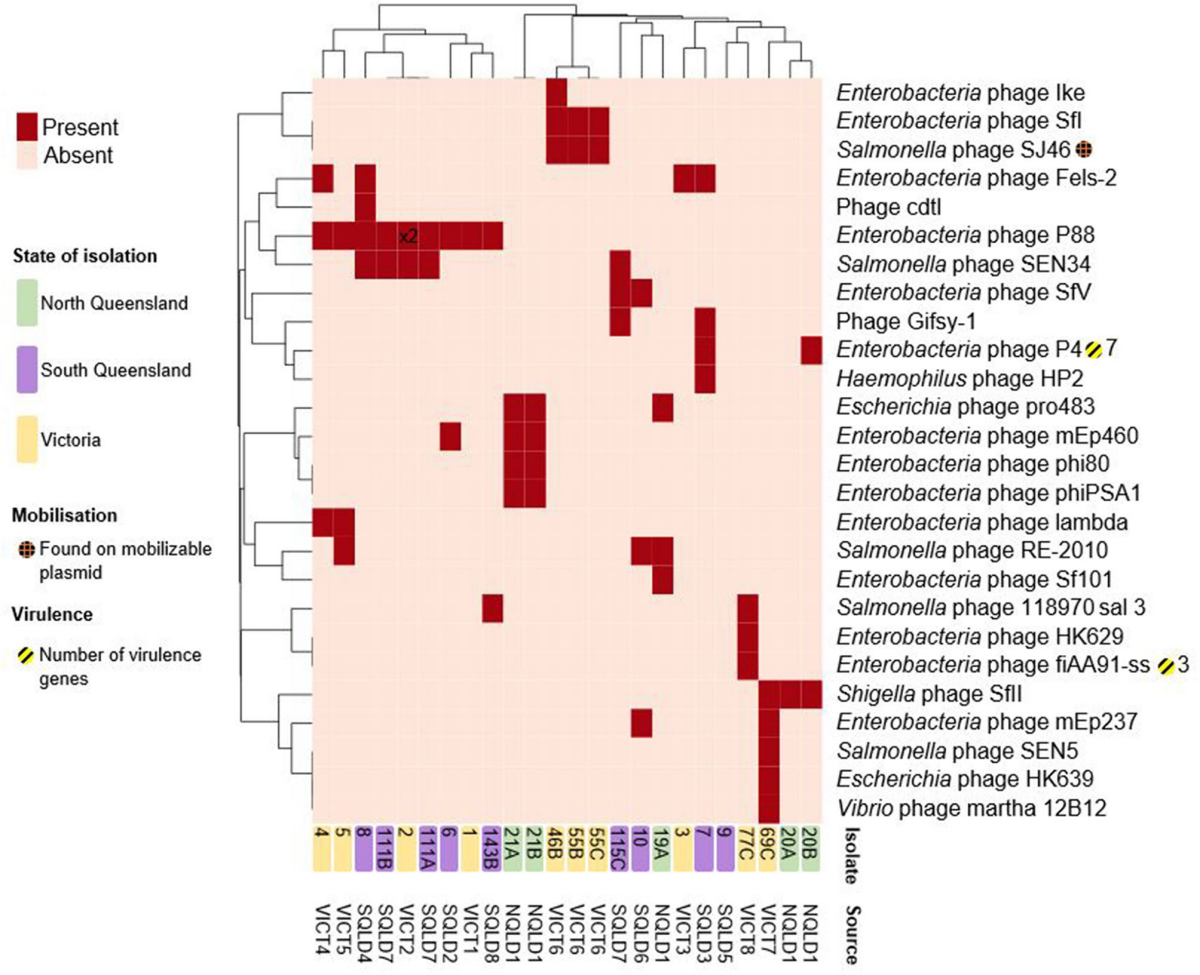
Heatmap of presence/absence of putatively intact prophages within the *E. coli* isolates. Clusters were based on correlation between prophages (x-axis) and isolates (y-axis). Dark red rectangles show hits to -like phages. Further information including accession numbers can be found in Supplementary Data Sheet 4. Clustering shown on rows and columns using the “correlation” distance measure. Victorian isolates are colored in yellow, North Queensland in green, South Queensland in purple. Presence on a putatively mobilizable plasmid represented by orange-hashed circles. Yellow-striped circles and the corresponding number represent presence and number of putative virulence factors. Number shown on heatmap (*x*^2^) indicate multiple intact prophages within the same genome. Heatmap produced using ClustVis.

The *E. coli* isolates contained a total of 26 distinct intact prophages (Figure 5, Supplementary Data Sheet 4). The *E. coli* 9 isolate was unusual in that it appeared to be missing several characteristics shared by all other strains. It lacked plasmids, the chromosomal *paaFGHIJKXY* locus, and intact chromosomal prophages. *Enterobacteria* phage P88-like prophage was the most commonly found present across a number of sites in both Victoria and Queensland, located in nine strains and twice within *E. coli* 2. There appears to be a limited local geographical correlation between intact prophages within strains at the farm level, while there is no discernible correlation across the 3 study regions. Three out of 3 VICT6 Victorian strains contained 2 prophages unique to them and 2/5 NQLD1 isolates shared 4 prophages. The *Salmonella* phage SJ46-like prophage (NC_031129) was found on the putatively mobilizable pCFSAN004177G_03-like plasmid in all three VICT6 isolates, while the remaining intact prophages were found on the chromosome (Supplementary Data Sheet 4).

These intact prophage regions were screened for virulence factors. Some prophages in *E. coli* have been shown to carry virulence factors (72). Amongst the 24 characterized isolates only two intact prophages had associated virulence factors. The *Enterobacteria* phage P4-like prophage (NC_001609), found in two Queensland isolates, carried the *fecIRABDE* and *yjhV* virulence-associated genes (Figure 5, Supplementary Data Sheet 4). The *Enterobacteria* phage fiAA91-ss-like prophage (NC_022750), found in one Victorian isolate, contained the *cdtABC* locus.

The genomes were interrogated to identify clustered regularly interspaced short palindromic repeats (CRISPR). CRISPR-Cas is a complex system that play a major role in host adaptive immune response and virulence (73, 74). They are found in most bacterial taxa, including *E. coli*, and also in most archaea. At least one sub-type of Cas1 gene (Cas-type I-E) was identified in 63% (15/24) isolates (Supplementary Data Sheet 7). All CRISPR-Cas systems were type I-E subtype (75), with 8 isolates across VICT, NQLD and SQLD containing intact Cas1, Cas2, Cas3, Cas5, Cas6, Cas7, Cse1, and Cse2. Isolate *E. coli* 1 only contained Cas2 and Cas3; signature genes that are associated with Cas1 and may not be functional due to lacking Cas1. The remaining nine isolates contained no CRISPR-Cas system genes. Among the isolates, we did not identify any Cas gene from six isolates from Victoria, one from South Queensland and 2 from North Queensland. The identified Cas from our study all belong to type 1-E sub-type. With exception to VICT6 isolate, all the remaining isolates without Cas1 gene had relatively higher virulence factors and moblisable elements (Supplementary Data Sheet 2, Figure 4). Although we did not directly determine the virulence or pathogenicity of isolates, our results are in agreement with the literature supporting the hypothesis of a negative correlation between the presence of CRISPR-Cas system and the number of predicted *E.coli* pathogenicity or virulence genes (76, 77). The absence or deletion of Cas1 in the 9 isolates we observed, indicates potential vulnerability to invaders or foreign nucleic acids, and possibly impaired chromosomal segregation (78).

**Figure 6:**
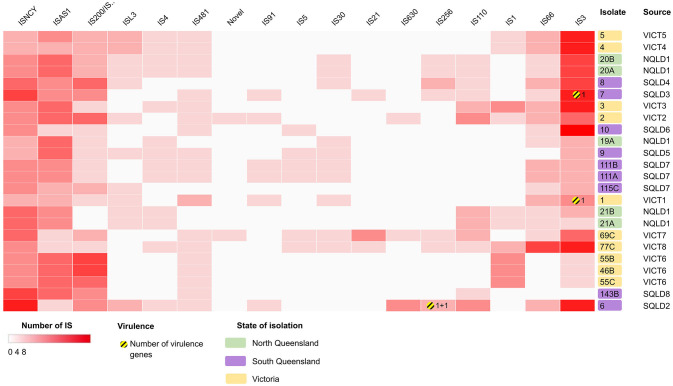
Heatmap showing the presence/absence of putatively intact insertion sequences (IS) within the *E. coli* isolates, clustered based on correlation between IS (x-axis) and isolates (y-axis). Darker red rectangles show more unique IS within the corresponding isolate. Further information can be found in Supplementary Data Sheet 5. Clustering shown on rows and columns using Euclidean correlation distance measure. Victorian isolates are colored in yellow, North Queensland in green, South Queensland in purple. Yellow-striped circles and the corresponding number represent presence and number of putative virulence factors. Heatmap produced using Clustergrammer.

The genomes were screened for the presence of transposons and insertion sequences (Figure 6, Supplementary Data Sheet 5). Isolates had 18.6 ± SD = 4.9 transposons within their genomes, with *E. coli* 6 having the most at 31, and *E. coli* 115C the fewest at 11. The most common family of transposons were the IS3 and ISNCY families, represented by ≥78 unique sequence across all isolates. There were few virulence factors associated with these transposable elements. *astA* and *east1* were associated with the individual IS256-family ISs in *E. coli* 6, *yjhV* was associated with a single IS3-family IS in *E. coli* isolates 1 and 7.

## Conclusions

The results in this study are consistent with prior genomic analysis of bovine mastitis-associated *E. coli* isolates in that there were only a few conserved core virulence factors, all of which are also present in commensal *E. coli* located within the bovine gastrointestinal tract. The comparison of mastitis strains collected from different geographical regions in Australia allowed the identification of common genes that could provide a partial explanation for the pathogenic effects in the mammary gland. Comparison of strains between regions demonstrated a high degree of similarity between isolates at the whole genome level. Analyses of the isolate genomes suggested there is no clear pathogenicity signature associated with the general metabolic, physiological, immunogenic, or resistance features of *E. coli* and the observed pathology in the *E. coli* infected mammary gland is not necessarily dependent on novel and unknown virulence factors specifically targeted at the mammary gland tissue. Culturing various strains of *E. coli* from clinical cases of bovine mastitis without reproducing disease in animal models has inherent risk. Koch's postulates are unfulfilled. Plasmids may be lost during the culture process directly affecting the resulting gene pool and subsequent conclusions from any analysis. Given there is likely no evidence for a bovine mastitis *E. coli* pathotype, alternative analyses such as *in vivo* RNA sequencing and metagenomics should be utilized to identify any relationships between gene content and bovine mastitis. Generation of such information would be invaluable for the development of diagnostic tools and provide opportunity for a greater understanding, prediction and improved decision making in the management of *E. coli*-associated bovine mastitis.

## Data Availability Statement

The datasets presented in this study can be found in online repositories. The names of the repositories and accession numbers can be found below:

https://www.ncbi.nlm.nih.gov/nuccore/JACCHD000000000
https://www.ncbi.nlm.nih.gov/nuccore/JACCHE000000000
https://www.ncbi.nlm.nih.gov/nuccore/JACCGV000000000
https://www.ncbi.nlm.nih.gov/nuccore/JACCGT000000000
https://www.ncbi.nlm.nih.gov/nuccore/JACCGS000000000
https://www.ncbi.nlm.nih.gov/nuccore/JACCGQ000000000
https://www.ncbi.nlm.nih.gov/nuccore/JACCGR000000000
https://www.ncbi.nlm.nih.gov/nuccore/JACCGN000000000
https://www.ncbi.nlm.nih.gov/nuccore/JACCGO000000000
https://www.ncbi.nlm.nih.gov/nuccore/JACCGP000000000
https://www.ncbi.nlm.nih.gov/nuccore/JACCGM000000000
https://www.ncbi.nlm.nih.gov/nuccore/JACCGL000000000
https://www.ncbi.nlm.nih.gov/nuccore/JACCHI000000000
https://www.ncbi.nlm.nih.gov/nuccore/JACCHF000000000
https://www.ncbi.nlm.nih.gov/nuccore/JACCHG000000000
https://www.ncbi.nlm.nih.gov/nuccore/JACCHC000000000
https://www.ncbi.nlm.nih.gov/nuccore/JACCHH000000000
https://www.ncbi.nlm.nih.gov/nuccore/JACCHA000000000
https://www.ncbi.nlm.nih.gov/nuccore/JACCHB000000000
https://www.ncbi.nlm.nih.gov/nuccore/JACCGZ000000000
https://www.ncbi.nlm.nih.gov/nuccore/JACCGY000000000
https://www.ncbi.nlm.nih.gov/nuccore/JACCGW000000000
https://www.ncbi.nlm.nih.gov/nuccore/JACCGX000000000
https://www.ncbi.nlm.nih.gov/nuccore/JACCGU000000000.

## Ethics Statement

The animal study was reviewed and approved by The University of Queensland Animal Ethics and National Guidelines.

## Author Contributions

JA: study design, sample collection, sample processing, and data analyses. BV: data analysis and interpretation. HA-H: sample collection and DNA extraction. TO: study design. HR: genome assembly and preliminary data analysis. AJ, MS, and RM: assisted with data interpretation. All authors contributed to drafting of the manuscript.

## Conflict of Interest

MS was employed by the company Terragen Biotech Pty Ltd. The remaining authors declare that the research was conducted in the absence of any commercial or financial relationships that could be construed as a potential conflict of interest.
